# Neighbor List Artifacts in Molecular Dynamics Simulations

**DOI:** 10.1021/acs.jctc.3c00777

**Published:** 2023-11-30

**Authors:** Hyuntae Kim, Balázs Fábián, Gerhard Hummer

**Affiliations:** † Department of Theoretical Biophysics, Max Planck Institute of Biophysics, Max-von-Laue Straße 3, 60438 Frankfurt am Main, Germany; ‡ International Max Planck Research School on Cellular Biophysics, Max-von-Laue Straße 3, 60438 Frankfurt am Main, Germany; ¶ Institute of Biophysics, Goethe University Frankfurt, Max-von-Laue-Straße 1, 60438 Frankfurt am Main, Germany

## Abstract

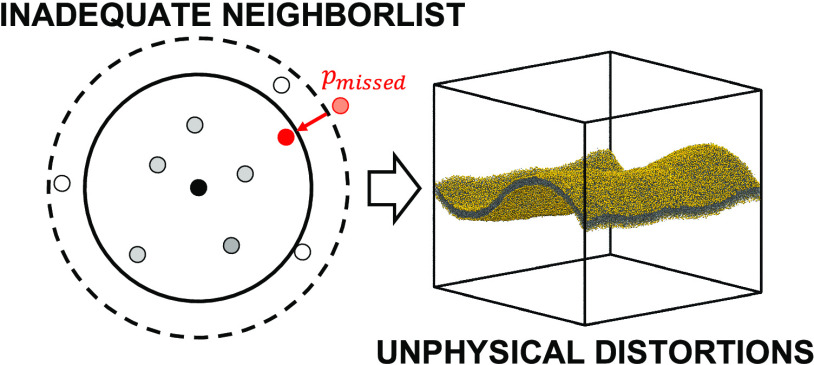

Molecular dynamics (MD) simulations are widely used in biophysical research. To aid nonexpert users, most simulation packages provide default values for key input parameters. In MD simulations using the GROMACS package with default parameters, we found large membranes to deform under the action of a semi-isotropically coupled barostat. As the primary cause, we identified overly short outer cutoffs and infrequent neighbor list updates that resulted in missed nonbonded interactions. Small but systematic imbalances in the apparent pressure tensor then induce unphysical asymmetric box deformations that crumple the membrane. We also observed rapid oscillations in averages of the instantaneous pressure tensor components and traced these to the use of a dual pair list with dynamic pruning. We confirmed that similar effects are present in MD simulations of neat water in atomistic and coarse-grained representations. Whereas the slight pressure imbalances likely have minimal impact in most current atomistic MD simulations, we expect their impact to grow in studies of ever-larger systems with coarse-grained representation, in particular, in combination with anisotropic pressure coupling. We present measures to diagnose problems with missed interactions and guidelines for practitioners to avoid them, including estimates for appropriate values for the outer cutoff *r*_l_ and the number of time steps nstlist between neighbor list updates.

## 1 Introduction

Molecular dynamics (MD) simulations are a powerful tool to probe molecular processes at a level of detail not currently accessible to experiments.^1^ The GROMACS molecular dynamics simulations package^2^ is widely used, in particular for applications in biophysics, chemistry, and soft-matter science. It is computationally efficient^2^ and easy to use with a wide range of atomistic and coarse-grained force fields quantifying the energetics of molecular interactions.^3,4^ Central to its high performance are the nearly linear scaling of the computational cost with system size and its efficient parallelization over multiple computational nodes.^5,6^ A key factor for the computational efficiency is the use of neighbor lists containing the pairs of interacting particles. To avoid costly neighbor list updates at every time step, the Verlet scheme includes a buffer of particles between the actual cutoff distance for pair interactions, *r*_c_, and an outer cutoff *r*_l_ > *r*_c_. The neighbor list is updated at time intervals chosen so that crossing from distances *r* > *r*_l_ to *r* < *r*_c_ by ballistic motion is highly unlikely. For the construction of neighbor lists on single-instruction multiple-data (SIMD) hardware architectures, GROMACS implements the MxN algorithm,^7,8^ which minimizes internode communication and memory footprint.^2,9^ The grouping of particles into spatial clusters by the MxN algorithm enables an efficient evaluation of the real-space pair interactions.

**Figure 1:**
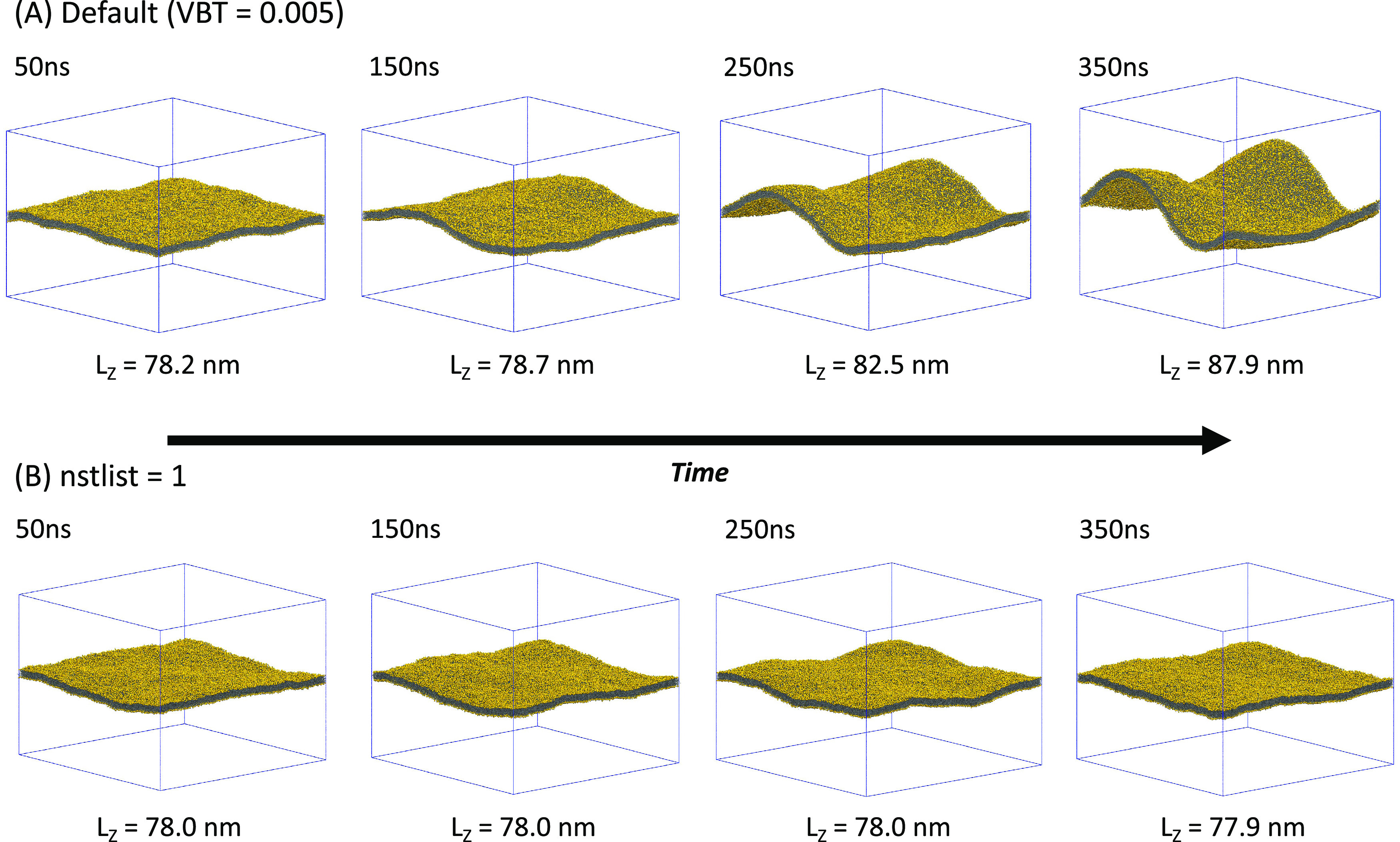
Large Martini POPC bilayer crumples in MD simulations with default simulation parameters, yet stays flat with frequent neighbor list updates. (A) Snapshots of the membrane (phosphate groups in gold) in MD simulation with default parameters. The Verlet-buffer-tolerance, which denotes the maximally allowed energy drift per particle between neighbor list updates due to missed nonbonded interactions, was set to VBT = 0.005 kJ·mol^–1^·ps^–1^; the outer cutoff was *r*_l_ = 1.269 nm; the number of time steps between neighbor list updates was nstlist = 25; and dual pair list was enabled. (B) Snapshots in an MD simulation with neighbor list updates enforced at every time step (nstlist = 1). Snapshots are at time points 50, 150, 250, and 350 ns (left to right). Simulation boxes are indicated as blue lines, and box heights *L_z_* are listed.

Here, we show that the use of default simulation parameters^10−12^ can cause artificial pressure oscillations and broken spatial isotropy. As a consequence, large membrane systems can undergo drastic deformations in the form of unrealistic buckling (Figure 1). We analyze the temporal evolution of lipid bilayers in the NPT ensemble with constant particle number *N*, pressure *P*, and temperature *T*; and of neat solvents in both NPT and NVT ensembles, the latter fixing the volume *V* instead of the pressure *P*. As the primary cause, we identify the infrequent construction of the neighbor list as a result of a somewhat too large update interval of nstlist time steps and a somewhat too short outer cutoff distance *r*_l_. Consequently, nonbonded interactions are occasionally missed in the force evaluation. The missed interactions cause errors in the elements of the instantaneous pressure tensor. In the NPT ensemble, these errors in the pressure lead to incorrect box rescaling by the barostat, both with the weak-coupling (Berendsen) barostat^13^ and the Parrinello–Rahman (PR) barostat.^14^ We conclude by providing tools that practitioners can use to detect such problems and guidance for minimizing their impact or avoiding them altogether.

The problems identified here may have afflicted earlier simulation studies. Pointedly, several studies of large membrane systems prevented excessive membrane undulations by restraining the vertical movement of certain lipid head groups with harmonic^15−17^ or flat-bottom^18−20^ potentials. One can also restrain the box with a weak harmonic potential, for example, by using the plumed software package^21^ as a GROMACS plug-in. However, the introduction of such external potentials is unsatisfactory, motivating our efforts to identify and correct the underlying issues.

## 2 Methods

### 2.1 Neighbor List and Missed Interactions

In MD simulations, nonelectrostatic nonbonded pair interactions are usually truncated beyond a given distance cutoff *r*_c_.^22^ Interactions beyond this cutoff are usually estimated analytically^23^ assuming a uniform density of particles outside the cutoff sphere, but can be evaluated in Fourier space for power–law potentials in a periodic system using the Ewald method as implemented, e.g., in the particle mesh Ewald (PME) algorithm.^24,25^ Without truncation of the real-space interactions, the computational cost of evaluating pairwise forces would scale with the square of the particle number *N* in the system. With a fixed cutoff *r*_c_, the cost scales roughly linearly with *N*. For the PME algorithm, evaluating the remaining long-range contributions results in *N* log *N* scaling.

The neighbor list of a particle contains the indices of its neighboring particles for which the pairwise interactions are explicitly evaluated in real space. In the Verlet scheme, the neighbor list is constructed by searching for neighbors within the cutoff radius *r*_l_, with *r*_l_ ≥ *r*_c_. The spherical shell between *r*_l_ and *r*_c_ provides a buffer so that neighbor list updates are not required at every time step. Neighbor search requires an evaluation of the pairwise distances, and hence, its computational cost scales at least linearly with the system size.^26^ Furthermore, the neighbor search requires internode communications, which can be a major bottleneck for modern hardware architectures.^10,27^ If neighbor list updates are performed only every nstlist time steps of length Δ*t* = dt, we expect that some pair interactions are missed because particle pairs move from distances *r* > *r*_l_ to *r* < *r*_c_ within the time interval nstlist × Δ*t*. For point particles of mass *m* uniformly distributed in space with number density ρ and moving with velocities following a Maxwell–Boltzmann distribution, we estimate (see Supporting Information (SI) text) the probability that a particular particle misses an interaction as
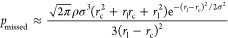
1where we assumed that nstlist, with *k*_B_ Boltzmann’s constant. For the analyses conducted in this study and in the figures, we instead used eq S8. In the SI Text, we extend the model from point particles to rigid and near-rigid molecules, such as TIP3P water, with an approximate treatment of rigid-body rotations.

### 2.2 GROMACS Input Parameters

In this study, we critically examine the following GROMACS input parameters: nstlist, nstenergy, nstcalcenergy, nstpcouple, nsttcouple, verlet-buffer-tolerance, and rlist, also denoted as *r*_l_. The parameters nstlist, nstenergy, nstcalcenergy, nstpcouple, and nsttcouple denote the number of time steps between neighbor list updates, energy sampling, energy evaluation, barostatting, and thermostatting, respectively. The default values recommended by the developers can be found in the manual:^12^nstlist = 10, nstenergy = 1000 and nstcalcenergy = 100.

The Verlet-buffer-tolerance (VBT) denotes the maximally allowed energy drift per particle between neighbor list updates due to missed nonbonded interactions. Its default value is 0.005 kJ·mol^–1^·ps^–1^.^2,12^ Changes in VBT result in adjustments of *r*_l_ and nstlist. In standard GROMACS runs, the values of *r*_l_ and nstlist are therefore not only system-dependent, but there is also no guarantee that the values are constant throughout a trajectory. In particular, the possible values of nstlist are 20, 25, 40, 50, 60, 80, and 100. The values of the adjusted *r*_l_ and nstlist can be found in the output log file. To ensure a constant value of nstlist, whether it is user-defined or the default value of 10, VBT must be disabled by setting VBT = −1.

In GROMACS, the maximally allowed energy drift, VBT, determines the frequency of neighbor list updates. By contrast, in LAMMPS^28^ the neighbor list is updated when any particle travels more than half the buffer thickness. As the system size increases, the time interval between updates shrinks to the point of forcing an update at every time step.^27^

### 2.3 MxN Algorithm and Dual Pair List

In SIMD hardware architectures, GROMACS employs the MxN algorithm for a grid-based neighbor search.^7,8^ The algorithm clusters a fixed number of particles by gridding the *xy* plane and binning along the *z* axis. The clusters with insufficient numbers of particles are filled with dummy particles. The implementation of the algorithm promises a high computational performance. Moreover, the clusters act as another layer of buffer on top of the predefined *r*_l_ – *r*_c_ shell, enabling a further increase in nstlist.^8^

The performance can be further improved by implementing a dual pair-list algorithm,^27^ using a long outer and a short inner list cutoff. The inner neighbor lists are generated from a pool of particles within the outer list and, hence, updated more frequently. The implementation of the dual pair-list algorithm reduces the overall computational cost of the neighbor search. The update frequencies and the cutoff radii for both the outer and inner list are by default controlled by VBT, and their exact values can be found in the log file. The dual pair-list algorithm can be disabled by setting VBT to −1. When the dual pair-list algorithm is enabled, *r*_l_ becomes the cutoff radius for the outer neighbor list. For GPUs, dynamic pruning is used to take advantage of their typically large execution width during the neighbor search.^27^

### 2.4 Membrane Bending Free Energy

The bending energy *E* associated with elastic deformations of a fluid and incompressible membrane can be estimated as an integral of the squared local mean curvature *H* over the membrane surface *A*(29)
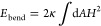
2where κ is the bending rigidity of the membrane. Here, we ignored the contribution of the Gaussian curvature, which is invariant for a given topology. We evaluated the local mean curvature *H* of the membrane systems using the MemCurv program^30^ and then integrated it numerically over the *xy* plane of the box, thus ignoring curvature corrections to the area element d*A*.

### 2.5 Simulation Code

Two versions of GROMACS were examined, namely, 2020.3 and 2023. All numerical analyses were performed using GROMACS 2020.3, the version for which the artifacts were initially observed. However, all of the system types described in this section were also simulated using GROMACS 2023, the latest version available. All the major artifacts caused by the use of inadequate combinations of *r*_l_ and nstlist were also observed in GROMACS 2023 runs with default parameters. These include the unphysical distortion of the large membrane systems, oscillations in the average instantaneous pressure, and broken spatial isotropy.

### 2.6 Simulation of Large and Small Martini Membranes

A large membrane system, consisting of 33,282 1-palmitoyl-2-oleoyl-glycero-3-phosphocholine (POPC) lipids and 6,721,594 water particles, was built using the insane.py^31^ script and the Martini force field (version 2.2).^4^ NaCl salt was added at a concentration of 0.15 M, and 10% of the water particles were replaced with the antifreeze beads (WF).^4^ The initial dimensions of the system were 100 nm × 100 nm × 80 nm. The system was equilibrated first in the NVT ensemble for 150 ns and then in the NPT ensemble with semi-isotropic pressure coupling for another 150 ns, using *r*_l_ = 1.422 nm and nstlist = 20. Production runs of 1 μs length were then performed using the *new-rf*(11) simulation parameters with *r*_c_ = 1.1 nm and a 20 fs time step. The system was coupled to a v-rescale thermostat^32^ at 310 K and semi-isotropically coupled to a PR barostat with a target pressure of 1 bar (τ_P_ = 12 ps). Also, note that nstcalcenergy = 1 was used for all the simulations here unless specified otherwise.

Similarly, a smaller Martini membrane system was built, consisting of 722 POPC lipids and 10,732 water particles. The corresponding initial box dimensions were 15 nm × 15 nm × 10 nm. All preparation procedures and input parameters were identical to those of the large membrane system, and it also underwent a 1 μs long production run.

### 2.7 Simulation of Water Systems

A system of neat Martini water was prepared for MD simulations in both the NVT and NPT ensembles. An initial volume of 6 nm × 6 nm × 6 nm contained 1530 Martini water particles. Similarly to the Martini membrane systems, the *new-rf*(11) simulation parameters with a 20 fs time step were used. Also, the ratio between water particles and antifreeze particles WF was set to 9:1. The system was equilibrated for 200 ns in both the NVT and NPT ensembles with *r*_l_ = 1.422 nm. Detailed analyses were performed on a few μs long production runs. We note that Martini water is, in effect, a Lennard-Jones (LJ) fluid lacking long-range electrostatics.

The distortion of the cubic box containing Martini water was studied in the NPT ensemble to examine the broken spatial isotropy due to the use of an inadequate combination of *r*_l_ and nstlist. A few varying conditions were examined, where the system was coupled to four different barostat types (semi-isotropically coupled PR, Berendsen and C-rescaling,^33^ and anisotropically coupled PR) with consistent target pressures of 1 bar. To detect the effects of possible systematic asymmetries in the calculated pressures as biased box distortions, we used the semi-isotropic and anisotropic pressure coupling schemes, even though these are not normally used to simulate isotropic solvent systems. Before the production runs, the equilibrated systems were isotropically scaled by factors of 0.99, 1.00, and 1.01, respectively. This scaling was intended to mimic possible volume artifacts caused by the use of inadequate combinations of *r*_l_ and nstlist. To account for possible anisotropy in the initial condition, the equilibrated systems were also rotated about the *x*, *y*, and *z* axes, respectively. This procedure was intended to eliminate any bias caused by the initial configuration of the system. For the same reason, the initial velocities of the particles were randomly generated, according to a Maxwell–Boltzmann distribution, for each of the 500 replicates, resulting in a sample size of 4500 runs for each barostat type. Then, the above procedures were performed using three different combinations: *r*_l_ = 1.9 nm, nstlist = 1; *r*_l_ = 1.9 nm, nstlist = 20; and *r*_l_ = 1.28 nm, nstlist = 25. Hence, 54,000 solvent simulations entered the statistical analysis of possible anisotropy. Finally, hypothesizing the asymmetric implementation of the MxN algorithm to be the cause of the potential anisotropy, we enforced the 1 × 1 atom pair list by recompiling the GROMACS MD engine with -DGMX_SIMD = None. We then performed 4500 replicate simulations of the fully anisotropically coupled system with *r*_l_ = 1.28 nm, nstlist = 25, and with 1 × 1 atom pair list setup.

Furthermore, the Martini water system was simulated in the NVT ensemble to illustrate that the various observed artifacts (except box rescaling) are not due to the barostat and occur independent of the ensemble type. As for the membrane systems, the respective production runs were 1 μs long. Except for the barostat settings, all input parameters were identical to those used for the NPT Martini water system.

Similarly, a cubic box containing pure TIP3P water^34^ was generated via CHARMM-GUI in the NVT ensemble,^35^ with dimensions of 5 nm × 5 nm × 5 nm. The system was equilibrated for 15 ns in both the NVT and NPT ensembles using *r*_l_ = 1.422 nm, while the production runs were 200 ns long with a 2 fs time step. The nonbonded interaction cutoff was *r*_c_ = 1.2 nm. The temperature was fixed at 310 K with the Nosé–Hoover thermostat.^36,37^ For the calculation of the power spectral density of the pressure in TIP3P water, we used the v-rescale thermostat^32^ to suppress the oscillatory contributions of the Nosé–Hoover thermostat. Finally, we used the PME algorithm with the default fourierspacing (0.12 nm).

### 2.8 Power Spectral Analysis

The power spectral density (PSD) of the scalar pressure was calculated using Welch’s method.^38^ We used the time series of the pressure as input, which were calculated and saved at every time step (nstenergy = 1). The resulting PSD was plotted as a function of the frequency in units of 1/(nstlist × Δ*t*). The visual inspection focused on peaks in the PSD as a means to identify characteristic time intervals of processes resulting in perturbations of the barostat action.

## 3 Results

### 3.1 Unphysical Distortion of the Large Martini Membrane

The temporal evolution of the large Martini membrane system simulated with default parameters and the PR barostat is shown in Figure 1A. Within the 350 ns of MD, the simulation box contracted in the *xy* membrane plane and expanded in the *z* direction. The box dimensions changed from 104.0 nm × 104.0 nm × 78.1 nm after equilibration to 97.1 nm × 97.1 nm × 89.7 nm at the end of the production run. This change in box shape left the overall volume approximately constant.

During the simulations, the large membrane buckled to form distinct folds in the *x* and *y* directions (Figure 1A). The potential energy and the enthalpy of the system increased by 13,200 and 24,600 kJ·mol^–1^, respectively, in 350 ns of MD. These increases are substantial also in relative terms, amounting to changes of ≈1.58 and ≈1.85%, respectively. Moreover, the membrane bending energy at the end of the MD simulation was estimated using eq 2 as *E*_bend_ ≈ 138 *k*_B_*T* ≈ 357 kJ·mol^–1^ for a bending rigidity of 25 *k*_B_*T*.^39,40^ The observed large increases in the system energy, the enthalpy, and the membrane bending energy strongly indicate that the observed deformation is unphysical.

**Figure 2:**
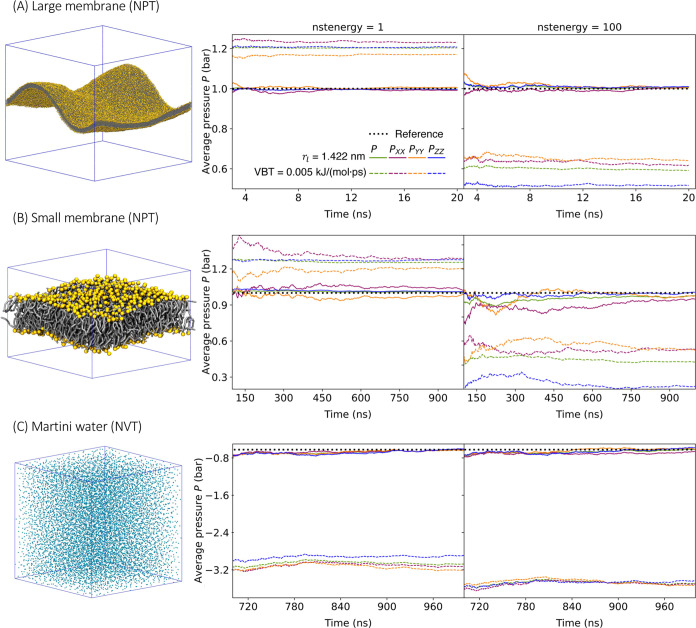
Pressure tensor elements deviate from the target pressure in MD simulations with the default cutoff handling. Running averages of the diagonal elements of the pressure tensor are shown for (A) the large and (B) the small Martini membrane systems in NPT MD simulations and (C) for the Martini water system in an NVT simulation. The left column shows snapshots of the systems. The center and right columns show the running averages evaluated every nstenergy = 1 and 100 steps, respectively. Results obtained with the default simulation parameters for cutoff handling (VBT = 0.005 kJ·mol^–1^·ps^–1^) are shown as dashed lines (see the legend for color). The solid lines show results for a larger outer cutoff *r*_l_ = 1.422 nm with nstlist = 20 fixed and dual pair list disabled. In (A, B), the target pressure of 1 bar in the NPT simulations is indicated by a dashed black line. For the NVT simulation in (C), the dashed black line indicates the consistent average obtained with *r*_l_ = 1.422 nm and nstlist = 20.

Pressure imbalances, not the barostats per se, appear to drive the box deformations. In MD simulations with semi-isotropically coupled PR (Figure 1A) and Berendsen barostats (Figure S1), we observed similar box deformations. Having thus ruled out an effect due to a specific barostat, we examined the components of the pressure tensor driving the barostat action. The running averages of the diagonal pressure tensor elements for the default setup (VBT = 0.005 kJ·mol^–1^·ps^–1^) are plotted as dashed lines in Figure 2A. Results are shown for the early phase of the simulation when the membrane is still flat (see Figure 1A). We observed that the three diagonal pressure tensor components deviate from the target pressure of 1 bar and each other.

### 3.2 Cutoff Handling Is Responsible for Membrane and Box Deformations

Differences in the apparent pressure average, as a function of the frequency of averaging, point to the underlying cause of the unphysical box distortions. The parameter nstenergy is the number of time steps between time points entering the pressure (and energy) averages. It should thus not affect the value of the average. However, for nstenergy = 1, we overestimated the pressure, and for nstenergy = 100, we underestimated it. These differences were significant and reproducible. Moreover, MD simulations of the small membrane system produced similar results (Figure 2B), pointing to the fact that we are dealing with a generic issue. As a possible explanation, we hypothesized that the pressure values calculated between neighbor list updates, which occur at intervals of nstlist = 20 or 25, differ from those right after neighbor list updates, with the former dominating the average for nstenergy = 1 and the latter for nstenergy = 100.

To test this hypothesis, we first performed MD simulations with nstlist = 1 to enforce neighbor list updates after every time step. As shown in Figure 1B, this eliminated the box and membrane deformations and made it possible to simulate a stable large membrane system without additional restraints. As a second test, we performed MD simulations in which we set a large outer cutoff distance of *r*_l_ = 1.422 nm together with neighbor list updates every nstlist = 20 time steps. As shown by the solid lines in Figure 2A, the pressure components then converged consistently to the target pressure of 1 bar for nstenergy = 1 and 100, respectively. Further support came from MD simulations of the small membrane system, where we also observed convergence to the target pressures (Figure 2B). In addition, MD simulations of a box of Martini water in the NVT ensemble, i.e., without membrane and barostat, showed in essence the same effect of apparent deviations between the pressure averages with default cutoff settings and nstenergy = 1 and 100, and consistent averages with *r*_l_ = 1.422 nm and nstlist = 20 (Figure 2C). Infrequent neighbor list updates thus emerged as a likely cause of pressure imbalances and associated box deformations.

### 3.3 Instantaneous Pressure Oscillates Between Neighbor List Updates

**Figure 3:**
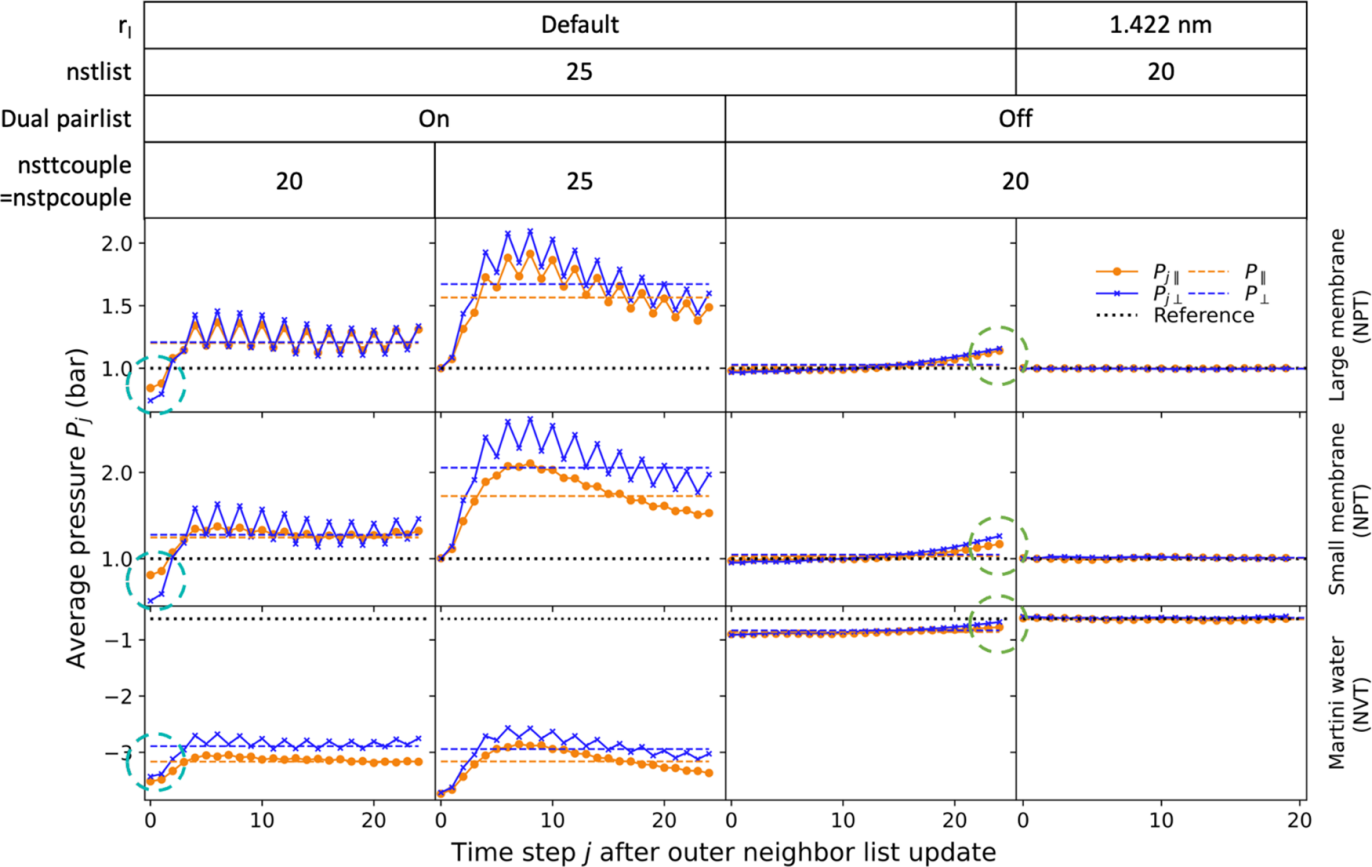
Average pressure tensor components deviate from the target pressure between neighbor list updates. Averages were performed over blocks of nstlist time steps starting immediately after a neighbor list update (time step 0). Results are shown for the large (top) and small (center) membrane systems in NPT simulations and for the NVT Martini water system (bottom). Results include the default cutoff setting with nstlist = 25 (three left columns) and the setting with *r*_l_ = 1.422 nm and nstlist = 20 (right column). The default *r*_l_ values for the NPT large and small membranes and the NVT solvent were 1.269, 1.267, and 1.28 nm, respectively. For the runs in column 3, the dual pair list was disabled. We averaged the lateral pressure components *P*_∥_ = (*P*_*xx*_ + *P*_*yy*_)/2 (orange curves) and showed the normal pressure as *P*_⊥_ = *P*_*zz*_ (blue curves). Dashed horizontal lines of matching colors denote the corresponding overall averages. Black horizontal dotted lines represent the reference pressure values. Cyan circles (left column) indicate deviations of the averaged pressures from the target. Green circles (column 3) indicate deviations just before the neighbor list update.

As a further test of the hypothesis that unresolved cutoff violations are at the heart of the observed problems, we examined the pressure as a function of the time between neighbor list updates (Figure 3). For the large and small membrane system in NPT ensembles and for the Martini water system in an NVT ensemble (top to bottom), we saved the time series of the pressure tensor components and averaged them over blocks of length nstlist starting immediately after a neighbor list update (time step 0). Results are shown for the default cutoff setting with nstlist = 25 (three left columns) and for the setting with *r*_l_ = 1.422 nm and nstlist = 20 (right column). We averaged the in-plane pressure components *P*_∥_ = (*P*_*xx*_ + *P*_*yy*_)/2 and show the normal pressure as *P*_⊥_ = *P*_*zz*_.

Consistent with our expectations, we found that with default cutoff settings, the average pressures immediately after a neighbor list update (i.e., at time step zero in Figure 3) are systematically lower than the pressures calculated between neighbor list updates (time steps > 0). However, we were initially puzzled by the observation that even at time step zero, the average pressure deviated from the target pressure (even though this is consistent with the findings in Figure 2 for nstenergy = 100). As a possible explanation, we considered that neighbor list updates came after nstlist = 25 time steps, yet thermostat and barostat actions after nsttcouple = nstpcouple = 20 time steps, and thus asynchronously (circles in the left column of Figure 3). Indeed, by setting nsttcouple = nstpcouple = nstlist = 25 time steps, the target and average pressures at time step 0 are consistent.

Counter to our expectations, we found the instantaneous pressure values to rise rapidly with time and to exhibit distinct oscillations. For missed interactions because of inadequate *r*_l_, we had expected a delayed and monotonic rise with time. As a source of this unexpected behavior, we identified the use of a dual pair list. When we disabled the dual pair-list evaluation by setting VBT = −1, both the rapid initial rise and the oscillations in the average pressures disappeared (column 3 in Figure 3). With the dual pair list enabled, the outer and the inner neighbor lists are maintained with two distinct intervals of 25 and 4 integration time steps, respectively. The zigzag oscillations in Figure 3 appear to be a superposition of two curves with these two periods. This argument is supported by simulations of Martini water in the NVT ensemble using a different hardware architecture, where the outer and inner lists were updated every 25 and 5 time steps, respectively (Figure S2). With the update intervals of the outer and the inner lists being multiples of five, the dominant oscillation period is also five time steps.

### 3.4 Pressure Deviations Correlate with Missed Particle Interactions

**Figure 4:**
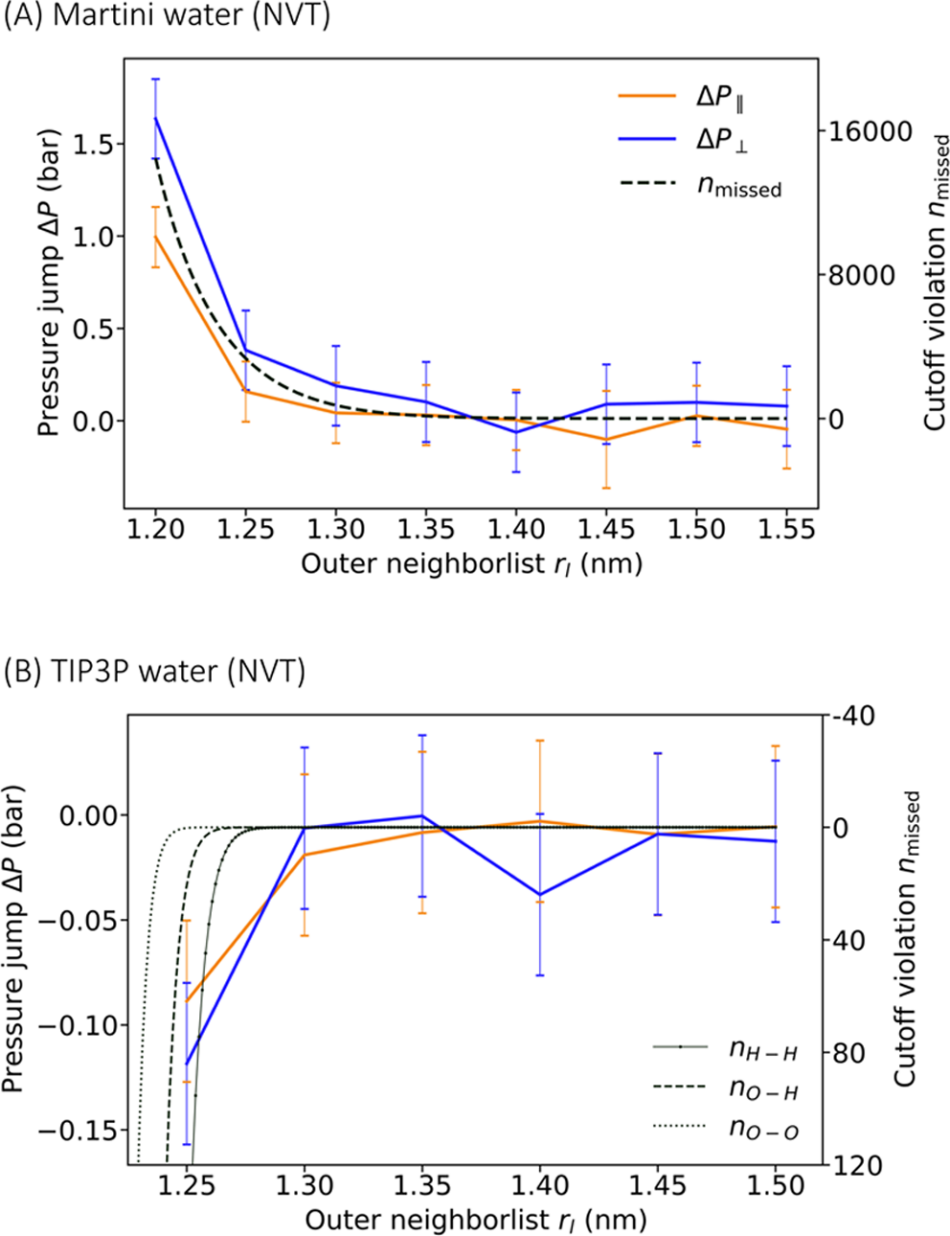
Differences Δ*P* in the pressures calculated just before and right after neighbor list updates follow the trend of missed interactions. Δ*P* (left scale) for lateral (orange) and normal pressures (blue) are shown as functions of *r*_l_ (A) for Martini water and (B) for TIP3P water, both simulated in NVT conditions with nstlist = 20 and dual pair list disabled. Standard deviations are shown as error bars. Also shown (right scale) is the expected number of missed interactions (black dashed lines). Considering the Martini water particles as point particles, the number of missed interactions (*n*_missed_) is evaluated using eq S8. For TIP3P water, the expected number of the missed hydrogen–hydrogen (*n*_H–H_), oxygen–hydrogen (*n*_O–H_), and oxygen–oxygen (*n*_O–O_) electrostatic interactions are evaluated using eqs S8 and S11.

In Figure 3, the average pressure values right after neighbor list updates are close to the target values. However, at the time step just before the neighbor list update (at time step 24 in Figure 3, column 3), we noticed significant positive deviations of Δ*P* from the pressure at time step 0, as indicated by green circles. In Figure 4, we plot these deviations for lateral (Δ*P*_∥_) and normal pressures (Δ*P*_⊥_) as functions of the outer cutoff *r*_l_ with the dual pair list disabled. Results are shown for Martini and TIP3P water. For reference, we also show *n*_missed_ for Martini water and *n*_H–H_, *n*_O–H_ and *n*_O–O_ for TIP3P water: *n*_missed_ is the expected mean number of unique cutoff violations of the water particles (eq S8); *n*_H–H_, *n*_O–H_ and *n*_O–O_ denote the expected number of unique cutoff violations of the hydrogen–hydrogen, oxygen–hydrogen, and oxygen–oxygen atom pairs, respectively, using eqs S8 and S11. Except for the shortest *r*_l_ ≈ *r*_c_, we find that the pressure errors just before neighbor list updates follow the trend of missed interactions. We note further that the Δ*P* errors are positive for Martini water, consistent with missed attractive Lennard-Jones interactions, as these lead to an underestimation of the cohesiveness. We thus conclude that missed interactions due to overly short outer cutoffs are the primary contributor to deviations Δ*P* in the pressure.

For TIP3P water, we found that the difference in the apparent pressure at time points just before and right after neighbor list updates tends to be negative, Δ*P <* 0 (Figures 4B and S3). The negative sign indicates that for TIP3P missed repulsive interactions dominate. From the rigid-rotor model described in the SI text, we indeed expect that at short times, the fast-moving hydrogen atoms with their low mass will result in missed repulsive hydrogen–hydrogen real-space electrostatic interactions. This is in contrast to Martini water, where Δ*P >* 0 results from missed attractive LJ interactions (Figure 4A). The nonmonotonic dependence of Δ*P* on *r*_l_ for TIP3P water (*r*_l_ ≲ 1.23 nm in Figure S3A and *r*_l_ ≲ 1.28 nm in Figure S3B) may be caused by a partial cancellation of contributions from missed attractive (opposite-charge and LJ) and repulsive (like-charge) interactions. Moreover, for small buffers *r*_l_ – *r*_c_ and long time intervals between the neighbor list updates nstlist × Δ*t*, the assumptions leading to eq S8 may no longer hold. A refined model could, for instance, use the estimated distribution of distances for missed pair interactions to estimate the impact on the virial.

### 3.5 Anisotropic Errors in Pressure Tensor Deform Box Shape

The errors Δ*P* in the pressure shown in Figure 4 tend to be somewhat anisotropic, even though the boxes had fixed cubic shape and volume. The lateral errors tend to be somewhat smaller than the normal errors, Δ*P*_∥_ < Δ*P*_⊥_. Therefore, we hypothesized that in MD simulations with both semi-isotropic and anisotropic barostats, we should see a tendency for the boxes to grow in the *z* direction. We tested this hypothesis by running repeated MD simulations of Martini water systems with semi-isotropic and fully anisotropic barostats of PR, Berendsen, and C-rescale type.

The results of these simulations confirm a tendency for the simulation box to expand preferentially along *z* by the action of the barostat (Table 1). This tendency is significant (*p*-value < 0.01 calculated from Pearson’s chi-squared test) for fully anisotropic pressure coupling with the PR barostat and two relatively poorer cutoff settings (*r*_l_ = 1.9 nm, nstlist = 20 and *r*_l_ = 1.28 nm, nstlist = 25). The observed tendency of the anisotropically coupled box to expand along *z* (Table 1) is consistent with the consistently larger error in the pressure along *z* seen in Figure 4. We note, however, that the direction of the box expansion is biased toward *z* but not deterministic. Spatial isotropy was restored when *r*_l_ = 1.9 nm and nstlist = 1 were used (*p*-value ≈ 0.89). For this setting, all interactions should be counted at all time steps, and the dual pair list is turned off.

**Table 1:** Spatial Isotropy Can be Compromised by the Cutoff Treatment in NPT MD Simulations of Martini Water^a^

	semi-isotropic			anisotropic
	Parrinello-Rahman	Berendsen	C-rescale	Parrinello-Rahman
	contraction: elongation			*X*: *Y*: *Z*
*r*_l_ (nstlist)	*p*-value			*p*-value
	1992:2508	2274:2226	2232:2268	1486:1489:1525
1.9 nm (1)	≪0.001	0.484	0.709	0.890
	1310:3190	1997:2503	2239:2261	1387:1536:1577
1.9 nm (20)	≪0.001	≪0.001	0.634	0.004
	1650:2850	1977:2523	2154:2346	1540:1322:1638
1.28 nm (25)	≪0.001	≪0.001	≪0.001	≪0.001
1.28 nm (25)				1496:1531:1473
1 × 1 pair list				0.768

a Four pressure coupling schemes were examined: semi-isotropically coupled PR, Berendsen, and C-rescale barostats, and an anisotropically coupled PR barostat. Four different combinations of *r*_l_ and nstlist were tested, including a 1 × 1 atom pair list (column 1). Columns 2–5 list the number of times the system elongated along a specific principal axis. *P*-values were calculated under the null hypothesis that the probabilities are equal to 1/2 for contraction and elongation along *z* in the semi-isotropic case, and equal to 1/3 for expansions along *x*, *y*, and *z* in the fully anisotropic case

Interestingly, we observed a statistically significant asymmetry in the box shape changes even for *r*_l_ = 1.9 nm and nstlist = 1 for MD simulations with a semi-isotropically coupled PR barostat (Table 1). Therefore, we checked alternative barostats with the same setting. For simulations with semi-isotropically coupled Berendsen and C-rescale barostats, the setting *r*_l_ = 1.9 nm and nstlist = 1 did not result in any significant bias in our tests (Table 1). Therefore, we suspect that the asymmetry in box shape changes with semi-isotropically coupled PR barostat may be a result of the specifics of the barostat implementation. For less stringent settings, *r*_l_ = 1.9 nm and nstlist = 20, we found that also the Berendsen barostat induced significant shape asymmetries but not the C-rescale barostat (Table 1). This further hints at the possibility that subtle differences in the barostat algorithm or implementation make the MD simulations susceptible to anisotropies in the pressure tensor. In practice, semi-isotropic coupling is typically used for systems with mechanical resistance, such as a lipid bilayer spanning the *xy* plane. For such systems, the consequences of the asymmetries detected here should be negligible.

A likely source of anisotropies is the asymmetric implementation of the MxN algorithm.^2,8^ The algorithm constructs the neighbor lists by gridding the *xy* plane and binning the particles along the *z* axis. Clusters with insufficient numbers of particles are filled with dummy particles, which have zero contribution on the cluster volume.^8^ Hence, the average cluster dimensions along the *z* axis would be smaller than those along the lateral axes. This effectively results in an anisotropic buffer thickness. Consequently, we expect that a larger fraction of cohesive interactions is missed along the *z* axis, which would explain the observed spatial anisotropies. To test this hypothesis, we enforced a 1 × 1 atom pair list (one particle per cluster) by recompiling the GROMACS MD engine with -DGMX_SIMD = None. We then performed 4500 replicate MD simulations of the fully anisotropically coupled system containing pure Martini water with *r*_l_ = 1.28 nm and nstlist = 25, as before. Consistent with our hypothesis, we found that by enforcing a 1 × 1 atom pair list, the asymmetry in the box shape changes disappeared (last row in Table 1).

## 4 Discussion

Incomplete neighbor lists resulting in missed pair interactions emerge as the primary cause of the various artifacts observed. Fast-moving particles occasionally cross from outside the outer cutoff, *r* > *r*_l_, to distances within the cutoff sphere, *r* < *r*_c_, of interaction partners between updates of the neighbor list. As a result, some nonbonded interactions are missed in the force evaluations at proceeding time steps up to the next neighbor list update. Affected are all interactions that rely on the neighbor list. This includes the real-space part of Coulomb interactions evaluated with the PME algorithm,^24^ as shown for TIP3P water. It should also affect the real-space part of other power–law interactions evaluated with the PME algorithm.^24,25^

As a partial fix to stabilize the simulations in cases where the outer cutoff *r*_l_ is too short, one can apply barostats (and thermostats) immediately after neighbor list updates and thus with all interactions accounted for. To leave room for efficiency optimizations for specific MD runs, the actual combination of *r*_l_ and nstlist in GROMACS is determined by the Verlet-buffer-tolerance VBT. In addition, GROMACS does not ensure that nsttcouple and nstpcouple are identical to nstlist by default. All barostat types in GROMACS act with a fixed frequency (nstpcouple) according to the instantaneous pressure tensor. If nstpcouple is not an integer multiple of nstlist and if the outer cutoff *r*_l_ is too short, then the barostat will at regular intervals operate with instantaneous pressures calculated with an incomplete list of pair interactions. In the case of Martini water, the missed interactions are attractive. Consequently, the barostat then acts to reduce the artificially high apparent pressure by increasing the system volume. However, such an increase in volume is artificial, the effect of which is only partially restored at those time points where barostat action immediately follows a neighbor list update. It is thus advantageous to set nsttcouple = nstpcouple = *n* × nstlist with integer *n* ≥ 1. Indeed, the unphysical distortion of the large Martini membrane system is greatly suppressed if nstpcouple = nstlist even with a relatively short outer cutoff of *r*_l_ = 1.269 nm (Figure S4). However, the undulation of the membrane shown in Figure S4 is still noticeably more pronounced than that simulated with nstlist = 1, resulting in a slightly larger *L_z_* = 78.2 nm, compared to *L_z_* = 77.9 nm in Figure 1B. Hence, setting nsttcouple = nstpcouple = nstlist in combination with default *r*_l_ alone may be insufficient.

Setting the outer cutoff *r*_l_ to values somewhat larger than the default further stabilizes the simulations. In numerical tests, we found it sufficient to obtain the default values of *r*_l_ and nstlist for the same system but with double the integration time step, and then to enforce these values manually together with nstpcouple = nstlist = nsttcouple. With VBT set to −1, this procedure also automatically disables dual pair list evaluation.

## 5 Recommendations for Practitioners

### 5.1 Diagnosis

Deviations between target and calculated pressures in NPT simulations serve as the primary indicator of possible issues with neighbor list construction and cutoff treatment. As shown in Figure 2, missed interactions as a result of an inadequate cutoff treatment tend to result in noticeable deviations between target and actual pressures and in small but again noticeable anisotropy as manifested by differences among the diagonal elements of the pressure tensor.

As a complication, the pressure and energy are calculated only at every nstcalcenergy time step, whereas neighbor lists are updated at every nstlist time step. If nstcalcenergy is an integer multiple of nstlist, the pressure tensor is always evaluated immediately after a neighbor list update. This synchrony can mask deviations (Figure 2B right). More frequent or asynchronous pressure calculation (e.g., by setting nstenergy = 1) can then reveal cutoff issues, as seen by comparing Figure 2B center and right. However, variations of nstlist along atomistic MD trajectories can further complicate the analysis.

Trial trajectories with pressures calculated every time step (nstenergy = 1) provide the basis for a more detailed analysis. Running averages (Figure 2) and averages over blocks of nstlist time steps (Figure 3) help to pinpoint problems with missed interactions and the resulting pressure imbalances.

**Figure 5:**
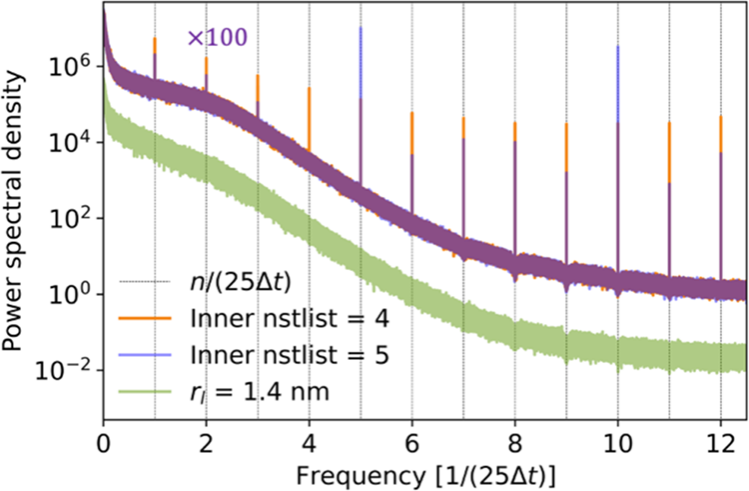
Power spectral density (PSD) of the scalar pressure calculated for Martini water in the NVT ensemble. Results are shown for *r*_l_ = 1.27 nm and nstlist = 25 (top curves; scaled by factor 100) with dual pair list and inner neighbor list updates every four (orange) and five (blue line) integration steps, respectively. Except for the oscillations at frequency multiples of 1/(nstlist × Δ*t*), the two curves nearly superimpose. Also shown (fluorescent green, bottom curve) is the PSD for a simulation with *r*_l_ = 1.422 nm and nstlist = 25 without dual cutoff, which does not show oscillations.

Oscillations in the scalar pressure as a result of neighbor list updates and barostat action can be revealed by a power spectral analysis. For an inadequate outer cutoff, the PSD of the scalar pressure shows distinct spikes at frequencies that are integer multiples of 1/(nstlist × Δ*t*), as shown in Figure 5. Their amplitude is modulated by the update frequency of the inner neighbor list. By enforcing a larger outer cutoff *r*_l_ with VBT set to −1 and without dual cutoff, these oscillations and the resulting features in the power spectral density disappear (lower curve in Figure 5). For the pressure in MD simulations of TIP3P water, we similarly observed distinct spikes in the PSD at integer multiples of 1/(nstlist × Δ*t*), which disappeared for large values of *r*_l_ and with the MxN algorithm disabled (Figure S5). Note that we used the v-rescale thermostat to avoid the oscillatory contributions to the pressure PSD in simulations with the Nosé–Hoover thermostat.^36,37^

### 5.2 Recommendations

For the existing software, pending updates, we recommend to manually define the outer cutoff *r*_l_ and the neighbor list update frequency nstlist as the default values obtained for the identical system with twice the time step. This requires setting verlet-buffer-tolerance = −1. For Martini systems using the *new-rf* parameters with nstlist = 20, *r*_l_ should be at least 1.35 nm. This can be achieved by setting VBT = ∼0.0002 kJ·mol^–1^·ps^–1^ (Figure S6). For atomistic systems with nstlist = 20, *r*_l_ should be at least 1.3 nm. Finally, nsttcouple and nstpcouple should be exact multiples of nstlist. However, these measures may significantly reduce the computational performance, especially for large systems. Depending on the hardware specifications, we observed a decrease in performance of up to ∼30%.

In the future, to minimize the impact on computational cost, a spatially isotropic neighbor search algorithm is desired also for the SIMD architecture. Spatial isotropy can for instance be restored to a significant degree with minimum computational overhead if the axis of the one-dimensional search is cycled through *x*, *y*, and *z* instead of keeping it fixed at *z*. More conservative default choices of *r*_l_ and nstlist will also help to ensure stable MD simulations. The choice of *r*_l_ and nstlist can be based on simple expressions for the probability of missed interactions such as eqs S8 and S11, including for atomistic MD simulations. Finally, all nstX variables (where X is energy, tcouple, pcouple, etc.) should be exact multiples of nstlist. In this way, energy output as well as thermostat and barostat actions benefit from freshly updated neighbor lists. If the dual pair list is enabled, the interval between updates of inner and outer neighbor lists should be set similarly to ensure that barostats, in particular, act with all interactions considered.

## 6 Concluding Remarks

The efficient calculation of pair interactions is at the heart of modern MD simulation codes. The construction of neighbor lists is a critical factor to reduce computational cost and to achieve near-linear scaling with system size, as well as efficient parallelization. We found that even a small number of missed nonbonded interactions can impact the accuracy and qualitative behavior of MD simulations. For large membrane systems, we observed that a slight but systematic error in the pressure with default settings for cutoff distance and neighbor list updates resulted in artificial membrane buckling caused by the counteraction of the barostat. We suspect that similar behavior motivated the imposition of restraints on lipid motion normal to the membrane in earlier studies.^15−20^ Slight but systematic anisotropies in the errors of the pressure result in anisotropic deformations of the box shape. We also observed distinct beating effects in the pressure time series. As underlying causes of these different but related problems, we identified (i) missed pair interactions as a result of too infrequent neighbor list updates, (ii) slight anisotropies in the neighbor list construction, and (iii) neighbor list and barostat updates at incommensurable time intervals. In most current MD simulations, in particular, at all-atom resolution, we expect the slight pressure imbalances to have minimal impact. However, as MD simulations are used to study ever-larger systems, such as the coarse-grained membrane systems in Figure 1, the issues will have to be addressed. Of particular concern are large systems with inherent anisotropies such as membrane systems or systems containing quasi-infinite molecules and molecular assemblies, including DNA and protein fibers spanning the periodic box, as these could become deformed by the action of the barostat in response to small but systematic errors in the pressure tensor components. Particular care should be taken in calculations of quantities related to stress or pressure differences such as interfacial tension and line tension. Whereas the immediate fixes of longer outer cutoff lengths *r*_l_ and disabled dual pair lists (VBT = −1) are associated with increased computational cost, we are confident that with the root causes identified, adjustments in algorithms and code can be made that will resolve the issues without major computational overhead.

## Supporting Information Available

Detailed derivation of number of missed interactions; the rigid-rotor model for missed interactions in systems with rigid or near-rigid molecules; power spectral density of pressure fluctuations in TIP3P water; the shape of the average pressure tensor between neighbor list updates depends on the update frequencies of the inner and outer list; difference Δ*P* in the pressure just before and right after a neighbor list update in the NVT TIP3P solvent system; in a simulation of the large Martini membrane system with nstpcouple = nstlist = 25, the unphysical box distortion is greatly suppressed; power spectral density (PSD) of the pressure fluctuations in MD simulations of TIP3P water in the NVT ensemble with nstlist = 20; probability *p*_missed_ of missed interactions (gray line) as function of the VBT parameter in GROMACS for the NVT Martini water system (PDF)

## References

[ref1] Gelpi J.; Hospital A.; Goñi R.; Orozco M. Molecular dynamics simulations: Advances and applications. Adv. Appl. Bioinf. Chem. 2015, 8, 37–47. 10.2147/AABC.S70333. PMC4655909 26604800

[ref2] Abraham M. J.; Murtola T.; Schulz R.; Páll S.; Smith J. C.; Hess B.; Lindahl E. GROMACS: High performance molecular simulations through multi-level parallelism from laptops to supercomputers. SoftwareX 2015, 1–2, 19–25. 10.1016/j.softx.2015.06.001.

[ref3] Lundborg M.; Lindahl E. Automatic GROMACS Topology Generation and Comparisons of Force Fields for Solvation Free Energy Calculations. J. Phys. Chem. B 2015, 119, 810–823. 10.1021/jp505332p. 25343332

[ref4] Marrink S. J.; Risselada H. J.; Yefimov S.; Tieleman D. P.; de Vries A. H. The MARTINI Force Field: Coarse Grained Model for Biomolecular Simulations. J. Phys. Chem. B 2007, 111, 7812–7824. 10.1021/jp071097f. 17569554

[ref5] Gecht M.; Siggel M.; Linke M.; Hummer G.; Köfinger J. MDBenchmark: A toolkit to optimize the performance of molecular dynamics simulations. J. Chem. Phys. 2020, 153, 144105 10.1063/5.0019045. 33086826

[ref6] Kutzner C.; Páll S.; Fechner M.; Esztermann A.; de Groot B. L.; Grubmüller H. Best bang for your buck: GPU nodes for GROMACS biomolecular simulations. J. Comput. Chem. 2015, 36, 1990–2008. 10.1002/jcc.24030. 26238484 PMC5042102

[ref7] Páll S.; Abraham M. J.; Kutzner C.; Hess B.; Lindahl E. Tackling Exascale Software Challenges in Molecular Dynamics Simulations with GROMACS. In Lecture Notes in Computer Science; Springer, 2015; pp 3–27.

[ref8] Páll S.; Hess B. A flexible algorithm for calculating pair interactions on SIMD architectures. Comput. Phys. Commun. 2013, 184, 2641–2650. 10.1016/j.cpc.2013.06.003.

[ref9] Buyya R.; Vecchiola C.; Selvi S. T. Principles of Parallel and Distributed Computing. In Mastering Cloud Computing; Morgan Kaufmann: Boston, 2013; Chapter 2, pp 29–70.

[ref10] Hess B.; Kutzner C.; van der Spoel D.; Lindahl E. GROMACS 4: Algorithms for Highly Efficient, Load-Balanced, and Scalable Molecular Simulation. J. Chem. Theory Comput. 2008, 4, 435–447. 10.1021/ct700301q. 26620784

[ref11] de Jong D. H.; Baoukina S.; Ingólfsson H. I.; Marrink S. J. Martini straight: Boosting performance using a shorter cutoff and GPUs. Comput. Phys. Commun. 2016, 199, 1–7. 10.1016/j.cpc.2015.09.014.

[ref12] Abraham M.; Alekseenko A.; Bergh C.; Blau C.; Briand E.; Doijade M.; Fleischmann S.; Gapsys V.; Garg G.; Gorelov S.; Gouaillardet G.; Gray A.; Irrgang M. E.; Jalalypour F.; Jordan J.; Junghans C.; Kanduri P.; Keller S.; Kutzner C.; Lemkul J. A.; Lundborg M.; Merz P.; Miletic V.; Morozov D.; Páll S.; Schulz R.; Shirts M.; Shvetsov A.; Soproni B.; van der Spoel D.; Turner P.; Uphoff C.; Villa A.; Wingbermühle S.; Zhmurov A.; Bauer P.; Hess B.; Lindahl E. GROMACS 2023.1 Manual, 2023 10.5281/zenodo.7852189.

[ref13] Berendsen H. J. C.; Postma J. P. M.; van Gunsteren W. F.; DiNola A.; Haak J. R. Molecular dynamics with coupling to an external bath. J. Chem. Phys. 1984, 81, 3684–3690. 10.1063/1.448118.

[ref14] Parrinello M.; Rahman A. Polymorphic transitions in single crystals: A new molecular dynamics method. J. Appl. Phys. 1981, 52, 7182–7190. 10.1063/1.328693.

[ref15] Ingólfsson H. I.; Melo M. N.; van Eerden F. J.; Arnarez C.; Lopez C. A.; Wassenaar T. A.; Periole X.; de Vries A. H.; Tieleman D. P.; Marrink S. J. Lipid Organization of the Plasma Membrane. J. Am. Chem. Soc. 2014, 136, 14554–14559. 10.1021/ja507832e. 25229711

[ref16] Larsen A. H. Molecular Dynamics Simulations of Curved Lipid Membranes. Int. J. Mol. Sci. 2022, 23, 8098 10.3390/ijms23158098. 35897670 PMC9331392

[ref17] Vögele M.; Köfinger J.; Hummer G. Hydrodynamics of Diffusion in Lipid Membrane Simulations. Phys. Rev. Lett. 2018, 120, 268104 10.1103/PhysRevLett.120.268104. 30004782

[ref18] Duboué-Dijon E.; Hénin J. Building intuition for binding free energy calculations: Bound state definition, restraints, and symmetry. J. Chem. Phys. 2021, 154, 204101 10.1063/5.0057845. 34241173

[ref19] Mori T.; Miyashita N.; Im W.; Feig M.; Sugita Y. Molecular dynamics simulations of biological membranes and membrane proteins using enhanced conformational sampling algorithms. Biochim. Biophys. Acta., Biomembr. 2016, 1858, 1635–1651. 10.1016/j.bbamem.2015.12.032. PMC4877274 26766517

[ref20] Kolossváry I.; Sherman W. Comprehensive Approach to Simulating Large Scale Conformational Changes in Biological Systems Utilizing a Path Collective Variable and New Barrier Restraint. J. Phys. Chem. B 2023, 127, 5214–5229. 10.1021/acs.jpcb.3c02028. 37279354 PMC10278137

[ref21] Tribello G. A.; Bonomi M.; Branduardi D.; Camilloni C.; Bussi G. PLUMED 2: New feathers for an old bird. Comput. Phys. Commun. 2014, 185, 604–613. 10.1016/j.cpc.2013.09.018.

[ref22] Frenkel D.; Smit B. Introduction. In Understanding Molecular Simulation: From Algorithms to Applications, 2nd ed.; Academic Press: San Diego, 2002; Vol. 1.

[ref23] Allen M. P.; Tildesley D. J. Computer Simulation of Liquids, 2nd ed.; Oxford University Press, 2017.

[ref24] Essmann U.; Perera L.; Berkowitz M. L.; Darden T.; Lee H.; Pedersen L. G. A smooth particle mesh Ewald method. J. Chem. Phys. 1995, 103, 8577–8593. 10.1063/1.470117.

[ref25] Sega M.; Dellago C. Long-range dispersion effects on the water/vapor interface simulated using the most common models. J. Phys. Chem. B 2017, 121, 3798–3803. 10.1021/acs.jpcb.6b12437. 28218854

[ref26] Panigrahy R. An Improved Algorithm Finding Nearest Neighbor Using Kd-trees. In Lecture Notes in Computer Science; Springer: Berlin Heidelberg pp 387–398.

[ref27] Páll S.; Zhmurov A.; Bauer P.; Abraham M.; Lundborg M.; Gray A.; Hess B.; Lindahl E. Heterogeneous parallelization and acceleration of molecular dynamics simulations in GROMACS. J. Chem. Phys. 2020, 153, 134110 10.1063/5.0018516. 33032406

[ref28] Thompson A. P.; Aktulga H. M.; Berger R.; Bolintineanu D. S.; Brown W. M.; Crozier P. S.; in ’t Veld P. J.; Kohlmeyer A.; Moore S. G.; Nguyen T. D.; Shan R.; Stevens M. J.; Tranchida J.; Trott C.; Plimpton S. J. LAMMPS - a flexible simulation tool for particle-based materials modeling at the atomic, meso, and continuum scales. Comput. Phys. Commun. 2022, 271, 108171 10.1016/j.cpc.2021.108171.

[ref29] Helfrich W. Elastic Properties of Lipid Bilayers: Theory and Possible Experiments. Z. Naturforsch. C 1973, 28, 693–703. 10.1515/znc-1973-11-1209. 4273690

[ref30] Bhaskara R. M.; Grumati P.; Garcia-Pardo J.; Kalayil S.; Covarrubias-Pinto A.; Chen W.; Kudryashev M.; Dikic I.; Hummer G. Curvature induction and membrane remodeling by FAM134B reticulon homology domain assist selective ER-phagy. Nat. Commun. 2019, 10, 2370 10.1038/s41467-019-10345-3. 31147549 PMC6542808

[ref31] Wassenaar T. A.; Ingólfsson H. I.; Böckmann R. A.; Tieleman D. P.; Marrink S. J. Computational Lipidomics with insane: A Versatile Tool for Generating Custom Membranes for Molecular Simulations. J. Chem. Theory Comput. 2015, 11, 2144–2155. 10.1021/acs.jctc.5b00209. 26574417

[ref32] Bussi G.; Donadio D.; Parrinello M. Canonical sampling through velocity rescaling. J. Chem. Phys. 2007, 126, 014101 10.1063/1.2408420. 17212484

[ref33] Bernetti M.; Bussi G. Pressure control using stochastic cell rescaling. J. Chem. Phys. 2020, 153, 114107 10.1063/5.0020514. 32962386

[ref34] Mark P.; Nilsson L. Structure and Dynamics of the TIP3P, SPC, and SPC/E Water Models at 298 K. J. Phys. Chem. A 2001, 105, 9954–9960. 10.1021/jp003020w.

[ref35] Jo S.; Kim T.; Iyer V. G.; Im W. CHARMM-GUI: A web-based graphical user interface for CHARMM. J. Comput. Chem. 2008, 29, 1859–1865. 10.1002/jcc.20945. 18351591

[ref36] Hoover W. G. Canonical dynamics: Equilibrium phase-space distributions. Phys. Rev. A 1985, 31, 1695–1697. 10.1103/PhysRevA.31.1695. 9895674

[ref37] Nosé S. A unified formulation of the constant temperature molecular dynamics methods. J. Chem. Phys. 1984, 81, 511–519. 10.1063/1.447334.

[ref38] Welch P. The use of fast Fourier transform for the estimation of power spectra: A method based on time averaging over short, modified periodograms. IEEE Trans. Audio Electroacoust. 1967, 15, 70–73. 10.1109/TAU.1967.1161901.

[ref39] Hernández-Muñoz J.; Bresme F.; Tarazona P.; Chacón E. Bending Modulus of Lipid Membranes from Density Correlation Functions. J. Chem. Theory Comput. 2022, 18, 3151–3163. 10.1021/acs.jctc.2c00099. 35389648 PMC9097289

[ref40] Eid J.; Razmazma H.; Jraij A.; Ebrahimi A.; Monticelli L. On Calculating the Bending Modulus of Lipid Bilayer Membranes from Buckling Simulations. J. Phys. Chem. B 2020, 124, 6299–6311. 10.1021/acs.jpcb.0c04253. 32597189

