# Supplementary material for: Neighbor List Artifacts in Molecular Dynamics Simulations
Source: J Chem Theory Comput. 2023 Nov 30;19(23):8919–29. doi: 10.1021/acs.jctc.3c00777 (PMC10720336; doi:10.1021/acs.jctc.3c00777)
Supplement: Supplementary file 1 — ct3c00777_si_001.pdf [file ct3c00777_si_001.pdf]

Supporting Information: Neighbor List Artifacts in Molecular Dynamics Simulations

Hyuntae Kim,[†,‡] Balázs Fábián,[†] and Gerhard Hummer[*,†,¶]

†*Department of Theoretical Biophysics, Max Planck Institute of Biophysics, Max-von-Laue Straße 3, 60438 Frankfurt am Main, Germany*

‡*International Max Planck Research School on Cellular Biophysics, Max-von-Laue Straße 3, 60438 Frankfurt am Main, Germany*

¶*Institute of Biophysics, Goethe University Frankfurt, Max-von-Laue-Straße 1, 60438 Frankfurt am Main, Germany*

E-mail: gerhard.hummer@biophys.mpg.de

Contents

1 SI Text **S2**

1.1 Detailed Derivation of Number of Missed Interactions S2

1.2 Rigid-Rotor Model for Missed Interactions in Systems with Rigid or Near-Rigid Molecules S5

1.3 Power Spectral Density of Pressure Fluctuations in TIP3P Water S6

2 Supplementary Figures **S7**

References **S11**

1 SI Text

1.1 Detailed Derivation of Number of Missed Interactions

In the following, we estimate the probability p that a pair of point particles initially at a distance $r_0 > r_l$ moves to a distance $r_t < r_c$ within a time t. In light of the short times relevant here, we assume purely inertial motion. However, because of the Gaussian nature of the propagated distribution of positions, the expressions generalize to diffusion dynamics, with a suitable re-definition of the width σ. We note that this derivation of the missed interactions is closely related to the estimation of the energy drift used by the GROMACS code to define r_l and `nstlist`.

For a Maxwell-Boltzmann distribution of velocities, the position distribution of particle 2 with mass m_2 at time t in the reference frame of particle 1 with mass m_1 is a 3D-Gaussian centered at the relative position at time 0 and with squared width $\sigma^2 = k_B\, T\, t^2\, (1/m_1 + 1/m_2)$. By integrating over the angles in spherical polar coordinates, we end up with the conditional

probability distribution of the pair distance at time t,

$$G(r_t|r_0) = \frac{r_t\, e^{-\frac{(r_0+r_t)^2}{2\sigma^2}} \left(e^{\frac{2r_0 r_t}{\sigma^2}} - 1\right)}{\sqrt{2\pi} r_0 \sigma} \tag{S1}$$

For simplicity, we assume that the positions of the two particles are uncorrelated and that the simulation box can be approximated by a sphere. The radius R of the sphere is chosen to match the volume of the simulation box, $V = 4\pi R^3/3$. The probability p for the particle pair to cross from a distance $r_0 > r_l$ to a distance $r_t < r_c$ is then,

$$p \approx \int_0^{r_c} dr_t \int_{r_l}^{R} dr_0\, 4\pi {r_0}^2 G(r_t|r_0)/V \tag{S2}$$

To estimate p, we first introduce reduced units $x = r_t/\sigma$, $y = r_0/\sigma$, $X = r_c/\sigma$, and $Y = r_l/\sigma$. Then, we set the upper integration limit for y to infinity, assuming that crossings of the buffer shell are only possible for pairs with initial distances close to r_l,

$$p \approx \frac{2\sqrt{2\pi}\sigma^3}{V} \int_0^X dx \int_Y^\infty dy\, x\, y\, e^{-(x+y)^2/2} \left(e^{2xy} - 1\right) \tag{S3}$$

The double integral can be evaluated analytically, resulting in

$$\begin{aligned} \frac{Vp}{\sigma^3} \approx{}& \frac{2\pi}{3}\left(2X^3 + (Y^3 - X^3)\,\mathrm{erf}\left(\frac{Y-X}{\sqrt{2}}\right) - (X^3+Y^3)\,\mathrm{erf}\left(\frac{X+Y}{\sqrt{2}}\right)\right) \\ &+ \frac{4\sqrt{2\pi}}{3} e^{-(X+Y)^2/2+XY} \left(XY \cosh(XY) + (X^2+Y^2-1)\sinh(XY)\right) \end{aligned} \tag{S4}$$

For $Y \gg X \gg 1$, the second error function evaluates to ≈ 1, and the hyperbolic functions cosh and sinh can be approximated by the dominant term $\exp(XY)/2$, and $X^2+XY+Y^2 \gg 1$. One then arrives at

$$\frac{Vp}{\sigma^3} \approx \frac{2}{3}\left(Y^3 - X^3\right)\left(\sqrt{2\pi}\frac{e^{-(Y-X)^2/2}}{Y-X} - \pi\,\mathrm{erfc}\left(\frac{Y-X}{\sqrt{2}}\right)\right) \tag{S5}$$

Using the asymptotic expansion of the complementary error function for large arguments z,

$$\sqrt{\pi}e^{z^2}\mathrm{erfc}(z) \sim \frac{1}{z} - \frac{1}{2z^3} \tag{S6}$$

we obtain

$$\frac{Vp}{\sigma^3} \approx \frac{2\sqrt{2\pi}\,(Y^2 + XY + X^2)}{3(Y-X)^2} e^{-(Y-X)^2/2} \tag{S7}$$

In a system of N identical particles of mass m and density $\rho = N/V$, we have $N(N-1)/2 \approx N^2/2$ distinct pair interactions. Using the full expression, eq S4, the total expected number of these missed pair interactions at time t after a neighbor list update is then,

$$\begin{aligned} n_{\text{missed}} &= Np_{\text{missed}} \approx \frac{N^2 p}{2} \\ &\approx \frac{\pi N \rho \sigma^3}{3}\left(\frac{2{r_c}^3}{\sigma^3} + \frac{{r_l}^3 - {r_c}^3}{\sigma^3}\,\mathrm{erf}\left(\frac{r_l - r_c}{\sqrt{2}\sigma}\right) - \frac{{r_l}^3 + {r_c}^3}{\sigma^3}\,\mathrm{erf}\left(\frac{r_l + r_c}{\sqrt{2}\sigma}\right)\right) \\ &+ \frac{2\sqrt{2\pi} N \rho \sigma^3 e^{-{r_l}^2 + {r_c}^2/2\sigma^2}}{3}\left(\frac{r_c r_l}{\sigma^2}\cosh\left(\frac{r_c r_l}{\sigma^2}\right) + \left(\frac{{r_c}^2 + {r_l}^2}{\sigma^2} - 1\right)\sinh\left(\frac{r_c r_l}{\sigma^2}\right)\right) \end{aligned} \tag{S8}$$

In the limit $p \ll 1$, we have

$$n_{\text{missed}} \approx Np_{\text{missed}} \approx \frac{N^2 p}{2} \approx \frac{\sqrt{2\pi}\rho\sigma^3 N\,({r_l}^2 + r_l r_c + {r_c}^2)}{3(r_l - r_c)^2} e^{-(r_l - r_c)^2/2\sigma^2} \tag{S9}$$

with p_{missed} as given in eq 1 of the main text. We note that σ is a function of t. We note further that for molecular systems such as water, the couplings arising from chemical bonds will interfere with these estimates already at short times, e.g., because of fast water rotation. In the following, we introduce an approximate formula for σ^2 aimed to interpolate between short-time and long-time rigid-body motion.

1.2 Rigid-Rotor Model for Missed Interactions in Systems with Rigid or Near-Rigid Molecules

So far, we estimated the probability of missed interactions by considering an ideal gas of point particles moving freely with velocities following a Maxwell-Boltzmann distribution. The distribution of displacement vectors for a particle of mass m in time t is then a 3D Gaussian with variance $\sigma^2 = k_B T t^2/m$. For rigid or near-rigid molecules, the situation is more complicated because both rotation and translation contribute to the motion. The free rotation of an anisotropic rigid body, while tractable analytically,[S1] is sufficiently involved to preclude the simple treatment of cutoff violations in the preceding section. However, at very short times each atom moves in effect freely, and at very long times we only have to consider the Gaussian displacement of the center of mass combined with a random distribution of each site on spherical shells about the center of mass. In the following, we describe a rigid-rotor model interpolating between the two limits.

For a site i of mass m_i in a molecule of total mass m_T, with a root-mean-squared distance $d_{i-C} = \langle|\mathbf{r}_i - \mathbf{r}_C|^2\rangle^{1/2}$ between site i and the center of mass, we define a linear rotor of length $d_{i-C}\, m_T/(m_T - m_i)$ with point masses m_i and $m_T - m_i$ at the two ends. The moment of inertia of the rotor is $I_i = m_i m_T {d_{i-C}}^2/(m_T - m_i)$. For angular velocities with a Maxwell-Boltzmann distribution, the distribution of the polar angle $\theta(t)$ travelled in time t with respect to the starting orientation is then a Gaussian with variance $t^2 k_B T/I_i$. It is thus tempting to approximate the distribution of site i about the starting point as a 3D Gaussian with width

$$ {\sigma_i}^2 = \frac{k_B T\, t^2}{m_T} + {d_{i-C}}^2 \left(1 - e^{-t^2/{\tau_i}^2}\right) \tag{S10} $$

where ${\tau_i}^2 = I_i/(k_B T)$ defines the square of a characteristic time for rotational motion. At short times, $t^2 \to 0$, we then recover the free motion of mass i, $\sigma_i \approx k_B T\, t^2/m_i$, as can be shown in a Taylor series expansion. At long times, $t \gg \tau_i$, the exponential term vanishes so that ${\sigma_i}^2$ is given by the sum of the free translational motion of the entire mass m_T plus

the mean-squared distance of site i from the center of mass. To estimate the missed pair interactions between sites i and j, we set

$$\sigma^2 = {\sigma_i}^2 + {\sigma_j}^2 \tag{S11}$$

in eq 1 of the main text and in eq S8. The number density ρ is, without loss of generality, that of sites j, and N in eq S8 is then the number of sites i. This Gaussian model should provide at least a rough approximation to assess the probability of missed particle interactions.

For TIP3P water, and similar water models with three massive sites, the distances of the hydrogen and oxygen atoms to the center of mass are $d_{\text{H-C}} = \sqrt{\left(d_{\text{OH}}\frac{m_{\text{O}}}{m_{\text{W}}}\right)^2 + \frac{{d_{\text{HH}}}^2}{4}\left(1 - \frac{{m_{\text{O}}}^2}{{m_{\text{W}}}^2}\right)}$ and $d_{\text{O-C}} = 2m_{\text{H}}d_{\text{O-HH}}/m_{\text{W}}$ with $d_{\text{O-HH}} = \sqrt{{d_{\text{OH}}}^2 - {d_{\text{HH}}}^2/4}$ and $m_{\text{W}} = m_{\text{O}} + 2m_{\text{H}}$. For the hydrogen site, the rotor has masses m_{H} and $m_{\text{H}} + m_{\text{O}}$, and for the oxygen site m_{O} and $2m_{\text{H}}$, with lengths of $d_{\text{H-C}}\, m_{\text{W}}/(m_{\text{O}} + m_{\text{H}})$ and $d_{\text{O-C}}\, m_{\text{W}}/(2m_{\text{H}})$, respectively. For simulations of TIP3P water, we use ${\sigma_{\text{HH}}}^2 = 2{\sigma_{\text{H}}}^2$, ${\sigma_{\text{OH}}}^2 = {\sigma_{\text{O}}}^2 + {\sigma_{\text{H}}}^2$, and ${\sigma_{\text{OO}}}^2 = 2{\sigma_{\text{O}}}^2$ in conjunction with eq S8 to estimate the number of HH, OH, and OO interactions, respectively, missed because of infrequent neighbor list updates.

1.3 Power Spectral Density of Pressure Fluctuations in TIP3P Water

Figure S5 shows the power spectral density (PSD) of the scalar pressure in MD simulations of TIP3P water in the NVT ensemble. The system was coupled to a v-rescale[S2] thermostat, as described in the Methods section. We fixed `nstlist` = 20. For $r_l = 1.25$ nm, the PSD shows distinct spikes at frequencies corresponding to integer multiples of $1/(\texttt{nstlist} \times \Delta t)$, consistent with the results for Martini water shown in Figure 5. For $r_l = 1.5$ nm, the spikes vanished. We conclude, first, that the PSD of the scalar pressure helps to identify issues with short outer cutoffs r_l and, second, that these issues can arise also in atomistic MD simulations.

2 Supplementary Figures

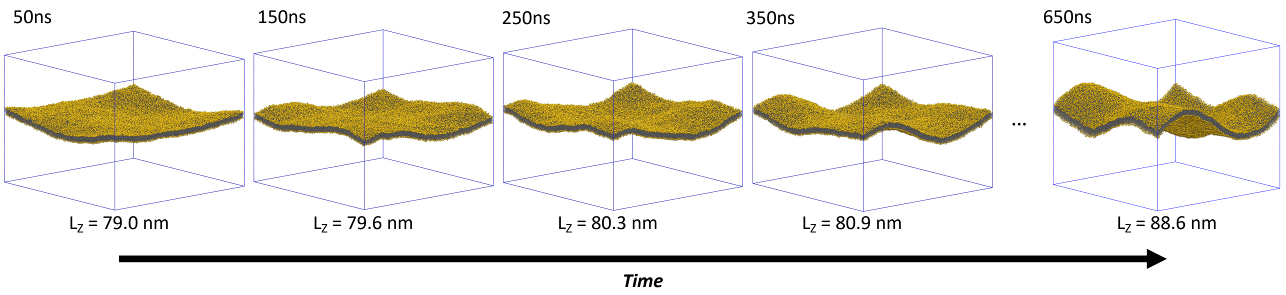

Figure S1: The large Martini POPC bilayer crumples in MD simulations with default simulation parameters (`VBT` $= 0.005$ kJ·mol^{-1}·ps^{-1}; $r_l = 1.27$ nm and `nstlist` $= 25$) and semi-isotropic coupling to a Berendsen barostat. Simulation details can be found in the Methods section. Snapshots of the lipid bilayer (phosphate groups in gold) in MD simulation at time points 50, 150, 250, 350, and 650 ns (left to right). Simulation boxes are indicated as blue lines and box heights are listed.

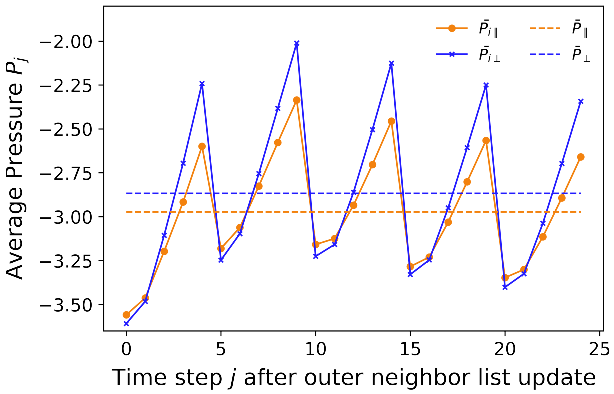

Figure S2: The shape of the average pressure tensor between neighbor list updates depends on the update frequencies of the inner and outer list. The average lateral pressure $P_{\|} = (P_{xx} + P_{yy})/2$ (orange curves) and normal pressure $P_{\perp} = P_{zz}$ (blue curves) are shown as function of the time step j after an update of the outer neighbor list. Results are shown for the NVT Martini water system with the default cutoff setting (`VBT` $= 0.005$ kJ·mol^{-1}·ps^{-1}; $r_l = 1.28$ nm and `nstlist` $= 25$) and with outer and inner neighbor list update intervals of 25 and 5, respectively. Simulation details can be found in the Methods section. Dashed horizontal lines of matching color denote the corresponding overall averages.

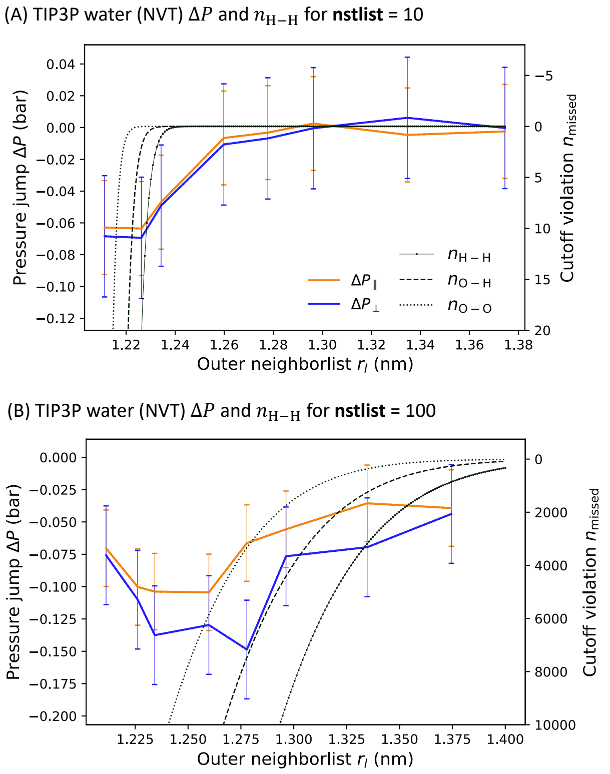

Figure S3: Difference ΔP in the pressure just before and right after a neighbor list update in the NVT TIP3P solvent system. ΔP for lateral (orange) and normal pressures (blue) is shown as function of r_l for (A) `nstlist` $= 10$ and (B) `nstlist` $= 100$. Dual pair list was disabled and standard deviations are shown as error bars. Also shown (right scale) are the expected number of missed hydrogen-hydrogen ($n_{\mathrm{H-H}}$), oxygen-hydrogen ($n_{\mathrm{O-H}}$) and oxygen-oxygen ($n_{\mathrm{O-O}}$) interactions (black lines), evaluated using eqs S4 and S11. For small buffers $r_l - r_c$ and long time intervals between the neighbor list updates `nstlist` $\times \Delta t$, the assumptions leading to eq S4 may no longer hold, resulting in non-monotonic dependence of ΔP on r_l for $r_l \lesssim 1.23$ nm in (A) and $r_l \lesssim 1.28$ nm in (B).

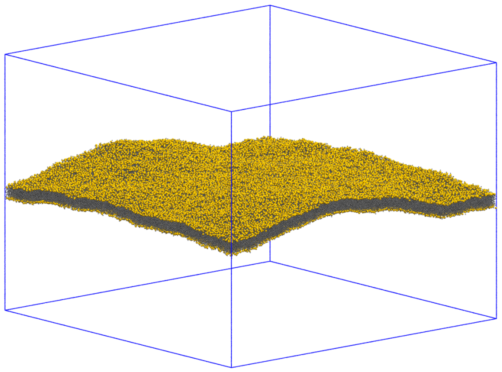

Figure S4: In a simulation of the large Martini membrane system with `nstpcouple` = `nstlist` = 25, the unphysical box distortion is greatly suppressed. The snapshot shows the membrane after a 1 µs production run. The vertical dimension of the box is 78.2 nm. However, despite the improvements the undulations of the membrane are noticeably more pronounced than with the more conservative `nstlist` = 1, which also resulted in a slightly shorter $L_z = 77.9$ nm (Figure 1B).

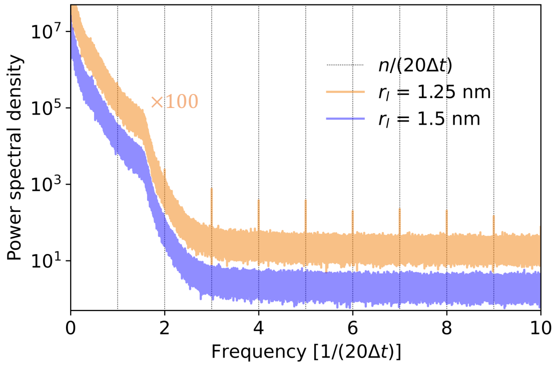

Figure S5: Power spectral density (PSD) of the pressure fluctuations in MD simulations of TIP3P water in the NVT ensemble with `nstlist` = 20. The peaks in the PSD at integer multiples of 1/(`nstlist` $\times \Delta t$) for $r_l = 1.25$ nm (orange; scaled by 100 for clarity) disappear for $r_l = 1.5$ nm (blue). The system was coupled to a v-rescale thermostat.

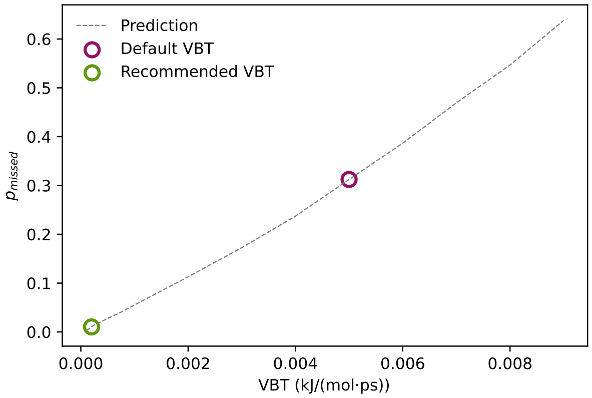

Figure S6: Probability p_{missed} of missed interactions (grey line) as function of the `VBT` parameter in GROMACS for the NVT Martini water system. Martini water was simulated with `VBT` $\in \{0.0001, 0.00025, 0.0005, 0.00075, 0.001, 0.002, 0.003, 0.004, 0.005, 0.006, 0.007, 0.008, 0.009\}$. The probability p_{missed} was then obtained from eq 1 for the respective values of r_l and `nstlist` listed in the output `log` files. For $0 <$ `VBT` < 0.005 kJ·mol^{-1}·ps^{-1}, the dependence is nearly linear. The default value `VBT` $= 0.005$ kJ·mol^{-1}·ps^{-1} is indicated by the magenta circle, and the recommended value of 0.0002 kJ·mol^{-1}·ps^{-1} by the green circle. The latter `VBT` value results in $r_l = 1.35$ nm and `nstlist` $= 20$.

References

(S1) van Zon, R.; Schofield, J. Numerical implementation of the exact dynamics of free rigid bodies. *J. Comput. Phys.* **2007**, *225*, 145–164.

(S2) Bussi, G.; Donadio, D.; Parrinello, M. Canonical sampling through velocity rescaling. *J. Chem. Phys.* **2007**, *126*, 014101.
